# Effects of repetitive transcranial magnetic stimulation on sequelae in patients with chronic stroke: A systematic review and meta-analysis of randomized controlled trials

**DOI:** 10.3389/fnins.2022.998820

**Published:** 2022-10-20

**Authors:** Gengbin Chen, Manfeng Wu, Tuo Lin, Guiyuan Cai, Jiayue Xu, Qian Ding, Wanqi Li, Cheng Wu, Hongying Chen, Yue Lan

**Affiliations:** ^1^Department of Rehabilitation Medicine, Guangzhou First People's Hospital, School of Medicine, South China University of Technology, Guangzhou, China; ^2^Postgraduate Research Institute, Guangzhou Sport University, Guangzhou, China; ^3^Department of Rehabilitation Medicine, The Second Affiliated Hospital, School of Medicine, South China University of Technology, Guangzhou, China; ^4^Guangzhou Key Laboratory of Aging Frailty and Neurorehabilitation, Guangzhou, China

**Keywords:** meta-analysis, rehabilitation, repetitive transcranial magnetic stimulation, sequelae, stroke, review

## Abstract

**Background:**

Stroke is the second leading cause of death worldwide, with a large proportion of survivors suffering from motor dysfunction and neuropsychiatric sequelae. Repetitive transcranial magnetic stimulation (rTMS) is a promising stroke rehabilitation intervention and is effective in improving neurological system function in stroke patients. In the current systemic review and meta-analysis, an overview of the most recent studies regarding the effectiveness of rTMS's potential to help chronic stroke patients recover from sequelae was provided.

**Methods:**

Relevant randomized controlled trials were retrieved from three online databases (Web of Science, Medline, and Embase). A total of 25 RCTs (*N* = 535 participants) were included. A meta-analysis was performed using a fixed-effects model or a random-effects model, and effect sizes were reported as weighted mean differences or standardized mean differences.

**Results:**

Administration of rTMS significantly improved upper limb function, hand function, and muscle tone in stroke patients throughout the chronic phase [≥6 months], but not lower limb mobility and strength. In terms of cognitive function, rTMS has a considerable positive impact on patients' cognitive performance. rTMS also alleviated apathy in stroke patients more than post-stroke depressive symptoms regarding mental functioning. Balance and walking function, as well as functional activities of daily living, of patients were dramatically improved by rTMS. However, the current conclusions should be taken carefully due to the small sample size of the meta-analysis.

**Conclusions:**

This is the first meta-analysis of rTMS treatment in patients with chronic stroke to inform the selection of the optimal treatment strategy for patients with chronic stroke, which demonstrated that rTMS treatment has the potential to improve the effects of sequelae by improving upper limb function, hand function, and muscle tone.

**Systematic review registration:**

https://inplasy.com/inplasy-2022-7-0095/, identifier: INPLASY202270095.

## Introduction

As the second leading cause of mortality worldwide (Lopez et al., 2006), stroke is also considered burdensome due to high morbidity rate, which leaves up to 50% of survivors with long-term disabilities (Donkor, 2018). Current estimates on the disease burden of stroke indicate that in 2010, there were roughly 71.7 million stroke survivors in sub-Saharan Africa (Moran et al., 2013). In 2017, there were 1.12 million strokes and 9.53 million stroke survivors in the European Union, and it is estimated that the number of stroke patients in the Europe will increase by 27% between 2017 and 2047 (Wafa et al., 2020). Additionally, 20–40% of stroke survivors develop spasticity (Zorowitz et al., 2013), 35% experience cognitive dysfunction (Tatemichi et al., 1994), and about one-third report melancholy, anxiety or apathy (Ferro et al., 2016). Worse still, a large proportion of stroke survivors struggle with ongoing disabilities and neuropsychiatric complications, which substantially lowers their quality of life.

Repetitive transcranial magnetic stimulation (rTMS) is a non-invasive therapeutic technique used for cortical excitability modulation. High frequency rTMS or intermittent theta burst stimulation (TBS) can increase cortical excitability, while low frequency rTMS or continuous TBS can decrease it (Pascual-Leone et al., 1998). According to the latest evidence-based guidelines (Lefaucheur et al., 2020) and reviews (Cantone et al., 2020) for rTMS treatment, rTMS has been applied in the areas of pain, dysphagia, limb movement, cognitive impairment, depression, etc. There have been several studies attempting to evaluate the effectiveness of rTMS for post-stroke rehabilitation, but a meta-analysis of how well it works for treating chronic complications post stroke has yet to be conducted. Since there is little scientific evidence supporting the effectiveness of rTMS in stroke survivors, we conducted a meta-analysis and systematic evaluation of evidence-based treatments to assess the data demonstrating that what the effects of rTMS on adult chronic stroke patients.

## Methods

### Protocol and search strategy

This meta-analysis followed the Preferred Reporting Items for Systematic Reviews and Meta-Analyses criteria (Moher et al., 2009) and is registered in the INPLASY International Platform for Registered Systematic Reviews and Meta-Analyses Protocols (registration number: INPLASY202270095).

A comprehensive literature search was conducted using three online databases (Web of Science, Medline, and Embase) in order to find relevant studies published in English from their inception until September 11, 2022. The search terms for the databases were revised and listed in Supplementary Table S1. In addition, the reference lists of the included papers were checked manually to identify any relevant studies.

### Eligibility criteria

The inclusion criteria for the study were as follows: (1) A randomized controlled trial (RCT) reporting the treatment outcomes of rTMS in adult chronic stroke patients; (2) all patients had stroke onset ≥6 months; (3) the intervention group received rTMS alone or in conjunction with other therapies, whereas the control group received sham rTMS (SrTMS) or no rTMS; and (4) minimum sample size was 5 patients.

Exclusion criteria were as follows: (1) Studies that could not meet the inclusion criteria; (2) crossover designs RCTs; and (3) studies published in non-English languages.

### Data extraction

Data was extracted and the quality of the eligible studies was evaluated separately by two investigators (CGB and WMF). If there were any discrepancies, a third independent investigator (CGY) was consulted. The information obtained from each study included the name of the first author, the year of publication, the number of participants, patient characteristics (age, gender, stroke type, mean time to stroke onset), treatment parameters (type of rTMS, intensity, number of pulses, duration and site of stimulation), control condition, outcome measures and adverse events. For each trial, the mean difference and standard deviation of the pre- and post-intervention outcome measures (rTMS and srTMS) were extracted for each group. For studies without numerical data, the authors were contacted to request the missing data or GetData Graph Digitizer 2.25 was employed for data extraction from the graphs based on the Cochrane Handbook for Systematic Reviews of Interventions (Higgins and Green, 2011).

### Data synthesis and analysis

Based on the outcomes of the included eligible studies, we conducted an analysis of seven aspects of motor dysfunction, speech and swallowing dysfunction, balance and walking dysfunction, cognitive dysfunction, psychological dysfunction, sensory dysfunction, and ADLs dysfunction remaining after chronic stroke, to determine the treatment effects of rTMS on these sequelae [≥6 months]. The analysis was performed following the recommendations for functional assessment of stroke rehabilitation according to the latest stroke guidelines (Teasell et al., 2020). For the post-stroke motor function, the results of upper extremity Fugl-Meyer Assessment, action research arm test, wolf motor function test, upper extremity Motricity Index and Manual Function test were used to assess upper limb motor function, and the results of the lower extremity Fugl-Meyer Assessment and lower extremity Motricity Index were used to assess lower limb motor function. Concurrently, the results of the box and block test, nine-hole peg test and purdue pegboard test were pooled to assess hand function, and the results of Grip strength and modified Ashworth Scale were used to assess post-stroke changes in strength and muscle tone. Results from the time up and go test and berg balance scale were pooled to assess post-stroke balance, and the results of the functional ambulation category and the 10-meter Walk Test and Gait speed were pooled to assess post-stroke walking. The mini mental status examination was used to assess post-stroke cognitive function and the level of psychological impairment post-stroke was assessed using the results of the Beck Depression Inventory, Hamilton Rating Scale for Depression, and quick inventory of depressive symptomatology. The results of the Functional Independence Measure, Barthel Index, Motor Activity Log and stroke impact scale were used to assess post-stroke ADLs. For the level of sensory dysfunction and speech and swallowing disorders, we performed only qualitative synthesis for these two levels because there are not enough studies to include them in the meta-analysis.

All statistical analyses were performed using the StataMP 14.0 software. Weighted mean differences (WMDs) and 95% CIs were used for studies using the same scoring system, and standardized mean differences (SMDs) were used for studies using different scoring systems, and these continuous data were pooled. The standardized mean differences (SMDs) or WMDs for changing scores (endpoints minus baseline scores) and their corresponding 95% confidence intervals (CIs) were used to assess the effect sizes of rTMS and controls. *I*^2^ statistic and Cochrane's Q test were used to assess heterogeneity among the included studies. If significant heterogeneity was observed (*I*^2^ > 50%, *P* < 0.05), a random-effects model was used. Alternatively, a fixed-effects model was used. A *P*-value of 0.05 was considered statistically significant. Sensitivity analysis was used to assess the stability of the system study.

Three subgroup analyses were performed to explore the factors affecting the functional recovery of the upper extremity in chronic phase stroke with rTMS: Stimulation frequency (low vs. high frequency); treatment site [Affected hemisphere primary motor cortex (AHM1) vs. Unaffected hemisphere primary motor cortex (UHM1)]; and number of stimulation pulses (600 pulses vs. 601-1200 pulses vs. >1,200 pulses).

### Quality assessment

The quality of the included studies was independently evaluated by two assessors using the PEDro scale (Maher et al., 2003; Blobaum, 2006). The scale consists of 11 elements, with each of the 10 quality criteria noted as 1 (pass) or 0 (fail). To determine the overall score for each study, the individual item scores were added. The maximum total score for each study was 10/10 because the first item is a gauge of external validity and is not taken into account when calculating overall findings. In addition, we used the Grading of Recommendations Assessment, Development, and Evaluation (GRADE) (Atkins et al., 2004) to determine the quality of the evidence provided by randomized controlled trials. This includes five criteria: risk of bias; inconsistency of results; imprecision of results; indirectness of evidence; and publication bias. Each piece of evidence was classified as high, medium, low or very low quality.

## Results

### Characteristics of the RCTs

**Figure 1:**
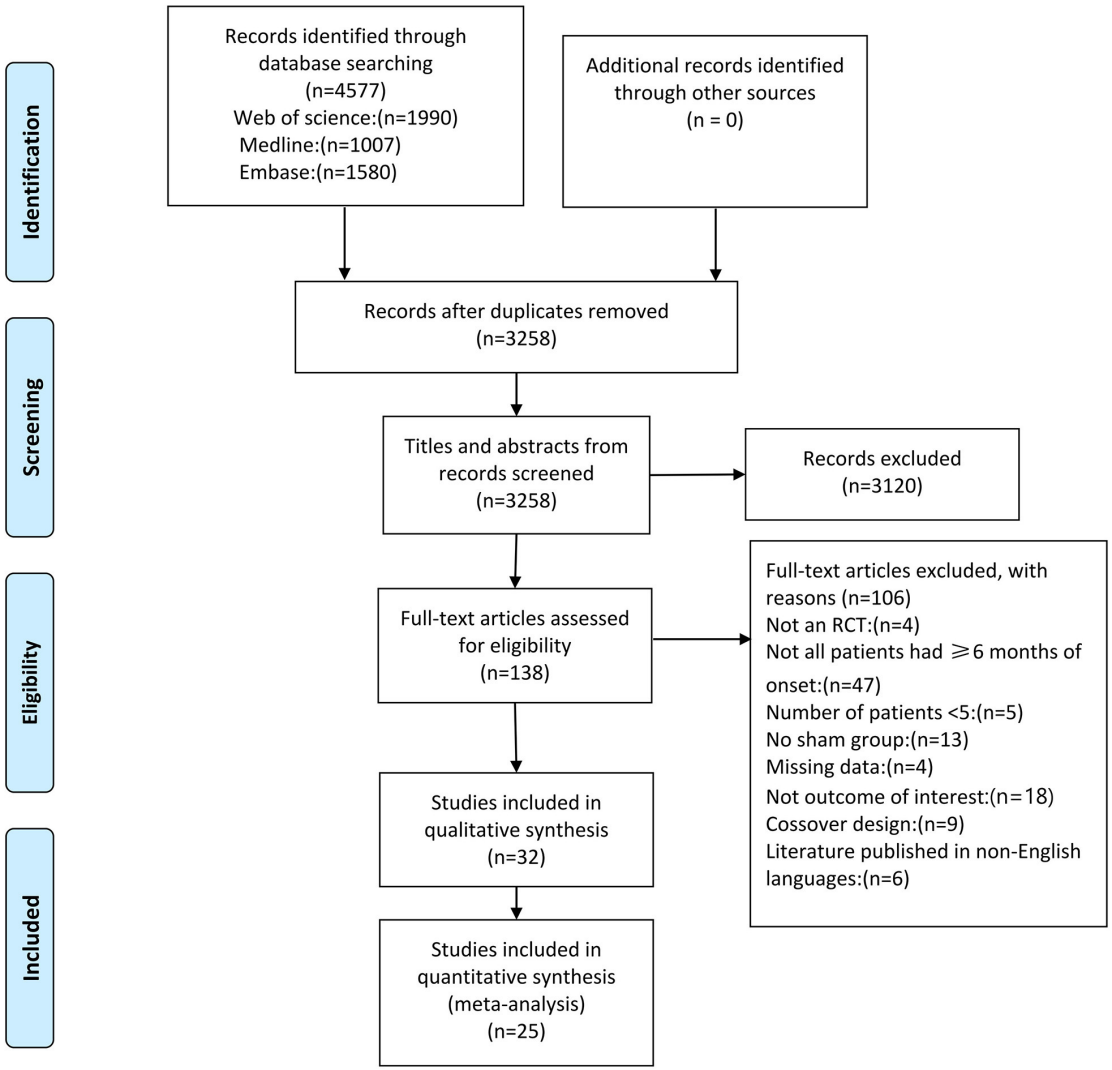
PRISMA flow chart on the selection and inclusion of studies.

The database search returned a total of 4,577 papers (Figure 1). Of these, 1,319 duplicate articles were excluded, and the remaining 3,258 papers were screened based on the title and abstract, resulting in the exclusion of 3,120 papers. For the remaining 138 full-text papers, eligibility was also assessed based on the inclusion criteria described previously. This led to the exclusion of another 106 studies that did not meet the eligibility criteria for the following reasons: non-RCT (4 studies); not all patients had ≥6 months of onset (47 studies); <5 patients (5 studies); lack of control group (13 studies); missing data (4 studies); not outcome of interest (18 studies); crossover design (9 studies), and non-English language publication (6 studies).

In total, 535 stroke patients from 25 high-quality RCTs were included in this meta-analysis and the detailed information of the included studies is shown in Supplementary Table S2. Sub-studies were identified in 2 studies (Kuzu et al., 2021; Zhang et al., 2022). This study included 2 experimental groups (Kuzu et al., 2021; Zhang et al., 2022) (different in terms of the rTMS protocol). As stated in the included studies, Most of the studies included patients with ischemic type stroke and patients with hemorrhage type stroke, except for the 7 studies (Higgins et al., 2013; Rose et al., 2014; Vongvaivanichakul et al., 2014; Wang et al., 2014; Ackerley et al., 2016; Lee and Cha, 2020; Hordacre et al., 2021) that did not report stroke type and 7 studies (Fregni et al., 2006; Di Lazzaro et al., 2013, 2016; Askin et al., 2017; Koch et al., 2019; Kuzu et al., 2021) that included only patients with ischemic stroke. The duration of stroke ranged from 6 months to 9 years and the duration of treatment ranged from 1 to 30 days. The proportion of female patients in the studies was 38%, except for 1 study (Vongvaivanichakul et al., 2014) that did not report a sex ratio. Patients in the experimental group varied in age from 52.4 to 74.0 years, whereas those in the control group were 52.6 to 71.0 years old. Of the assessment methods in this meta-analysis, all were subjective clinical scales, except for gait speed and Grip strength, which were measured with objective instruments. A total of 9 of the 27 studies used 1 Hz rTMS (Fregni et al., 2006; Wang et al., 2012; Higgins et al., 2013; Barros Galvão et al., 2014; Rose et al., 2014; Vongvaivanichakul et al., 2014; Askin et al., 2017; Dos Santos et al., 2019; Kuzu et al., 2021) with 600-1,500 pulses per session. Nine studies used high frequency rTMS ranging from 5 Hz (900 pulses) (Wang et al., 2019; Lee and Cha, 2020), 10 Hz (700-3,000 pulses) (de Oliveira et al., 2014; Gu and Chang, 2017; Sasaki et al., 2017; Jeong et al., 2020; Liu et al., 2020; Hordacre et al., 2021) to 20 Hz (2,000 pulses) (Malcolm et al., 2007). Nine studies used TBS with frequencies ranging from intermittent TBS (600-1,200 pulses) (Ackerley et al., 2016; Chen et al., 2019; Koch et al., 2019; Lin et al., 2019; Zhang et al., 2022), continuous TBS (600 pulses) (Di Lazzaro et al., 2013, 2016; Kuzu et al., 2021) to a combination of continuous and intermittent TBS (600 pulses) (Zhang et al., 2022). Almost all studies used sham coils, tilt coils, 20% RMT stimulation intensity or sham coils without stimulator output for sham stimulation, one study (Askin et al., 2017) had no sham rTMS, and one study (Jeong et al., 2020) did not describe the details of sham rTMS.

Based on the evaluation with the PEDro score, the quality scores of the included studies ranged from 6 to 10, and the average quality score of the included studies was 8.32 ± 0.85 (mean ± standard deviation), indicating a high methodological quality (Table 1). Eighty-four percent of the 25 randomized controlled trials did not use concealed allocation. This was followed by no blinded therapists (*n* = 17), no blinded subjects (*n* = 2) and no blinded assessors (*n* = 2). The quality of the evidence evaluated using the GRADE methodology is illustrated in Supplementary Table S3.

**Table 1:** Assessment of risk of bias in the included studies.

**References**	**Criteria**											**Total**
	**1**	**2**	**3**	**4**	**5**	**6**	**7**	**8**	**9**	**10**	**11**	
Ackerley et al. (2016)	Y	1	1	1	1	1	1	1	1	1	1	10
Askin et al. (2017)	Y	1	0	1	0	0	1	1	1	1	1	7
Barros Galvão et al. (2014)	Y	1	1	1	1	0	1	1	1	1	1	9
Chen et al. (2019)	Y	1	0	1	1	0	1	1	1	1	1	8
de Oliveira et al. (2014)	Y	1	0	1	1	1	1	1	1	1	1	9
Di Lazzaro et al. (2016)	Y	1	0	1	1	0	1	1	1	1	1	8
Di Lazzaro et al. (2013)	Y	1	0	1	1	0	1	1	1	1	1	8
Dos Santos et al. (2019)	Y	1	0	1	1	0	1	1	1	1	1	8
Fregni et al. (2006)	Y	1	0	1	1	1	1	1	1	1	1	9
Gu and Chang (2017)	Y	1	0	1	1	1	1	1	1	1	1	9
Higgins et al. (2013)	Y	1	0	1	1	0	1	1	1	1	1	8
Hordacre et al. (2021)	Y	1	0	1	1	0	1	1	1	1	1	8
Jeong et al. (2020)	Y	1	0	1	0	0	0	1	1	1	1	6
Koch et al. (2019)	Y	1	0	1	1	0	1	1	1	1	1	8
Kuzu et al. (2021)	Y	1	0	1	1	0	1	1	1	1	1	8
Lee and Cha (2020)	Y	1	0	1	1	1	1	1	1	1	1	9
Lin et al. (2019)	Y	1	0	1	1	1	1	1	1	1	1	9
Liu et al. (2020)	Y	1	1	1	1	0	1	1	1	1	1	9
Malcolm et al. (2007)	Y	1	0	1	1	0	1	1	1	1	1	8
Rose et al. (2014)	Y	1	0	1	1	1	1	1	1	1	1	9
Sasaki et al. (2017)	Y	1	0	1	1	1	1	1	1	1	1	9
Vongvaivanichakul et al. (2014)	Y	1	0	1	1	0	0	1	1	1	1	7
Wang et al. (2012)	Y	1	1	1	1	0	1	1	1	1	1	9
Wang et al. (2019)	Y	1	0	1	1	0	1	1	1	1	1	8
Zhang et al. (2022)	Y	1	0	1	1	0	1	1	1	1	1	8

Criteria numbers: 1, eligibility criteria; 2, random allocation; 3, concealed allocation; 4, similar groups at baseline; 5, blinding subjects; 6, blinding therapists; 7, blinding assessors; 8, outcome obtained in more than 85% of the subjects; 9, intention-to-treat analysis; 10, between-group statistical comparisons; 11, point estimates and measures of variability.

### Effects of rTMS on the recovery of motor function in chronic stroke patients

**Figure 2:**
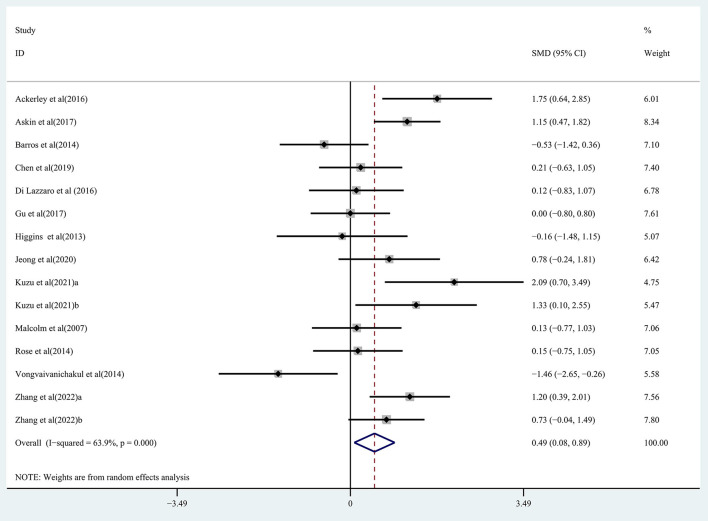
Forest plot of the effect of rTMS treatment on upper extremity function recovery in chronic stroke patients.

**Figure 3:**
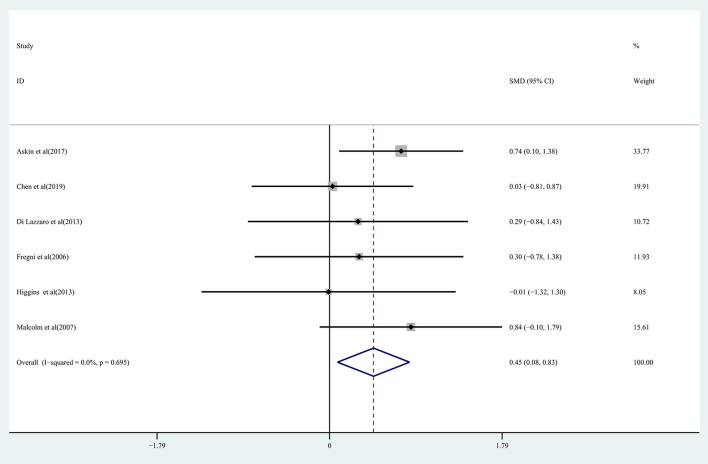
Forest plot of the effect of rTMS treatment on hand function recovery in chronic stroke patients.

Pooled data from 15 studies (Malcolm et al., 2007; Higgins et al., 2013; Barros Galvão et al., 2014; Rose et al., 2014; Vongvaivanichakul et al., 2014; Ackerley et al., 2016; Di Lazzaro et al., 2016; Askin et al., 2017; Gu and Chang, 2017; Chen et al., 2019; Jeong et al., 2020; Kuzu et al., 2021; Zhang et al., 2022) was employed to determine the effects of rTMS on the upper extremity movement in chronic stroke patients, and low-quality evidence showed results that demonstrated a significant improvement in the treatment group (SMD: 0.49; 95% CI: 0.08–0.89; *P* = 0.019) (Figure 2), but relatively high heterogeneity (*I*^2^ = 63.9%, *P* < 0.001). Regarding the effect of rTMS on hand function, pooled data from six studies (Fregni et al., 2006; Malcolm et al., 2007; Di Lazzaro et al., 2013; Higgins et al., 2013; Askin et al., 2017; Chen et al., 2019) was used, and moderate quality evidence observed a significant improvement in the treatment group (SMD: 0.45; 95% CI: 0.08-0.83; *P* = 0.017) (Figure 3), with 0% heterogeneity.

**Figure 4:**
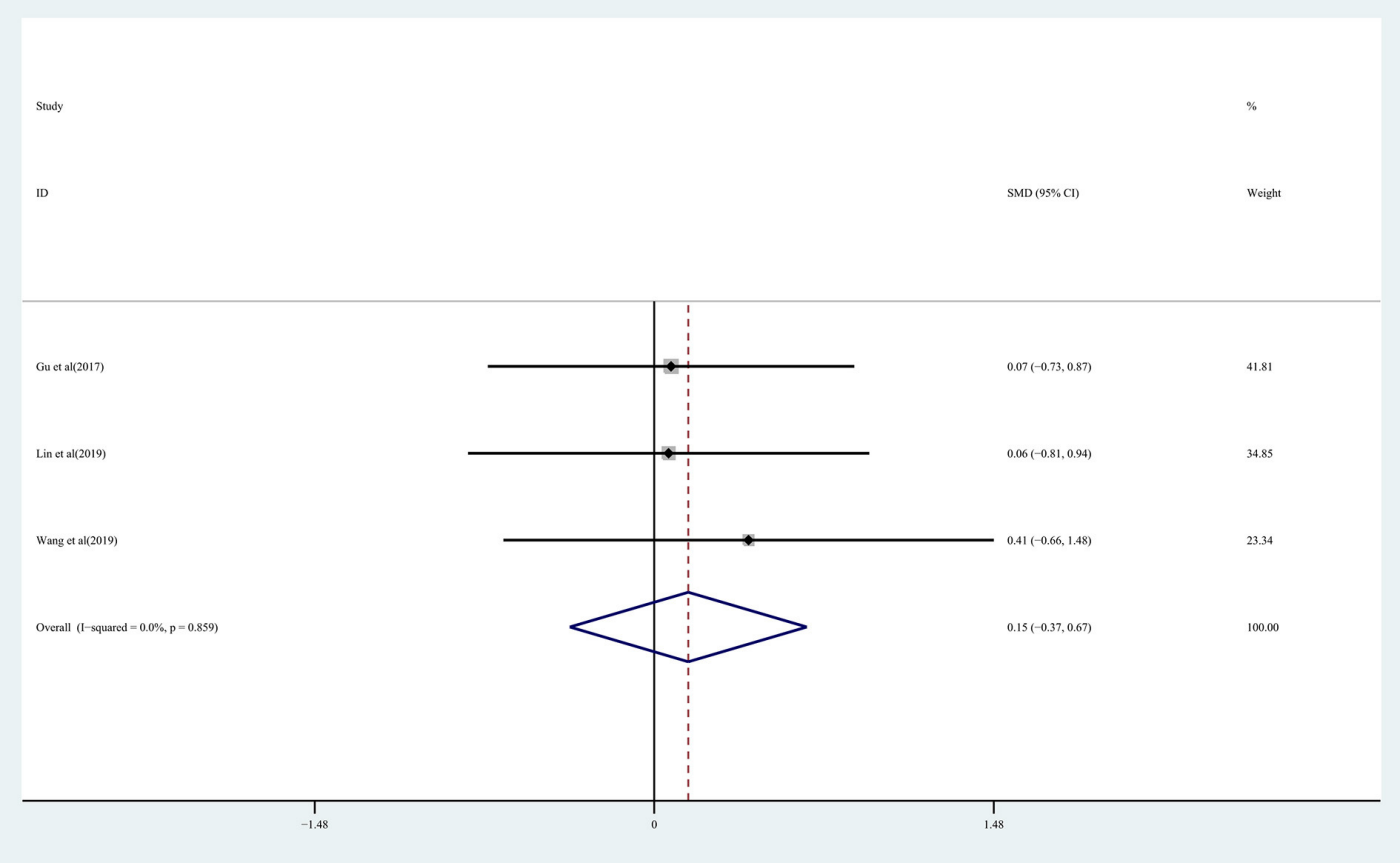
Forest plot of the effect of rTMS treatment on lower extremity function recovery in chronic stroke patients.

The effect of rTMS on lower extremity motor function in chronic stroke patients was assessed by pooling data from three studies (Gu and Chang, 2017; Lin et al., 2019; Wang et al., 2019) with no significant improvement being detected in the treatment group (SMD: 0.15; 95% CI: −0.37 to 0.67; *P* = 0.573) (Figure 4), with 0% heterogeneity.

**Figure 5:**
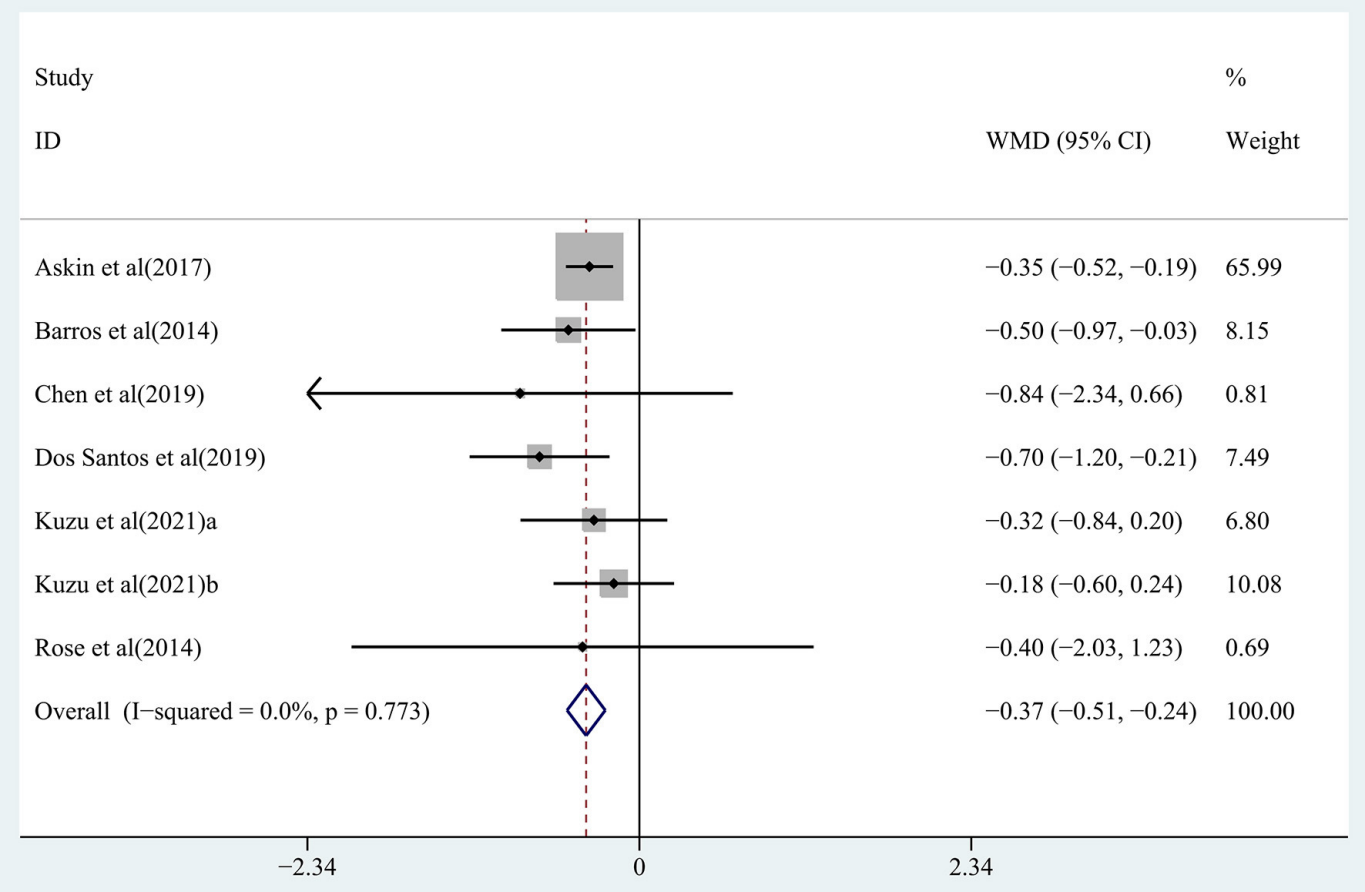
Forest plot of the effect of rTMS treatment on muscle tone reduction in chronic stroke patients.

The effect of rTMS on muscle tone change and strength recovery in chronic stroke patients was assessed by pooling data from seven studies (Barros Galvão et al., 2014; Rose et al., 2014; Askin et al., 2017; Chen et al., 2019; Dos Santos et al., 2019; Kuzu et al., 2021) and four studies (Di Lazzaro et al., 2013; Higgins et al., 2013; Rose et al., 2014; Jeong et al., 2020), respectively, and the pooled data showed moderate quality evidence of a significant reduction in muscle tone in the treatment group (WMD, −0.37; 95% CI: −0.51 to −0.24; *P* = 0) (Figure 5), with 0% heterogeneity. There was no significant improvement in strength recovery (WMD, 0.00; 95% CI: −0.15 to 0.15; *P* = 0.998) in the treatment group (Supplementary Figure S1), with 0% heterogeneity.

For rTMS for upper limb function in patients with chronic-phase stroke, subgroup analysis based on stimulation frequency did not find a significant effect for low-frequency (SMD: 0.20, 95% CI: −0.71 to 1.11, *P* = 0.667, *I*^2^ = 79.6%, *P* < 0.001) and high-frequency stimulation (SMD: 0.24, 95% CI: −0.27 to 0.76, *P* = 0.358, *I*^2^ = 0%, *P* = 0.474; Supplementary Figure S2A) had significant effects. Subgroup analysis based on treatment site found a significant effect for Affected hemisphere M1 (SMD: 0.57, 95% CI: 0.12 to 1.02, *P* = 0.013, *I*^2^ = 30.5%, *P* = 0.207) but a significant effect for Unaffected hemisphere M1 (SMD: 0.35, 95% CI: −0.47 to 1.18, *P* = 0.405, *I*^2^ = 77.7%, *P* < 0.001; Supplementary Figure S2B) was not found. Studies were classified by the number of stimulus pulses (600 pulses vs. 601–1,200 pulses, vs. 1,200 pulses). The subgroup of 600 pulses showed a significant effect size (SMD: 0.75, 95% CI: 0.17–1.32, *P* = 0.011, *I*^2^ = 44.6%, *P* = 0.125), but 601–1,200 pulses (SMD: 0.40, 95% CI: −0.24 to 1.04, *P* = 0.218, *I*^2^ = 72.4%, *P* = 0.001) and >1,200 pulses did not (SMD: 0.10, 95% CI: −1.18 to 1.39, *P* = 0.875, *I*^2^ = 72.2%, *P* = 0.058) (Supplementary Figure S2C).

### Effect of rTMS on cognitive recovery in chronic stroke patients

**Figure 6:**
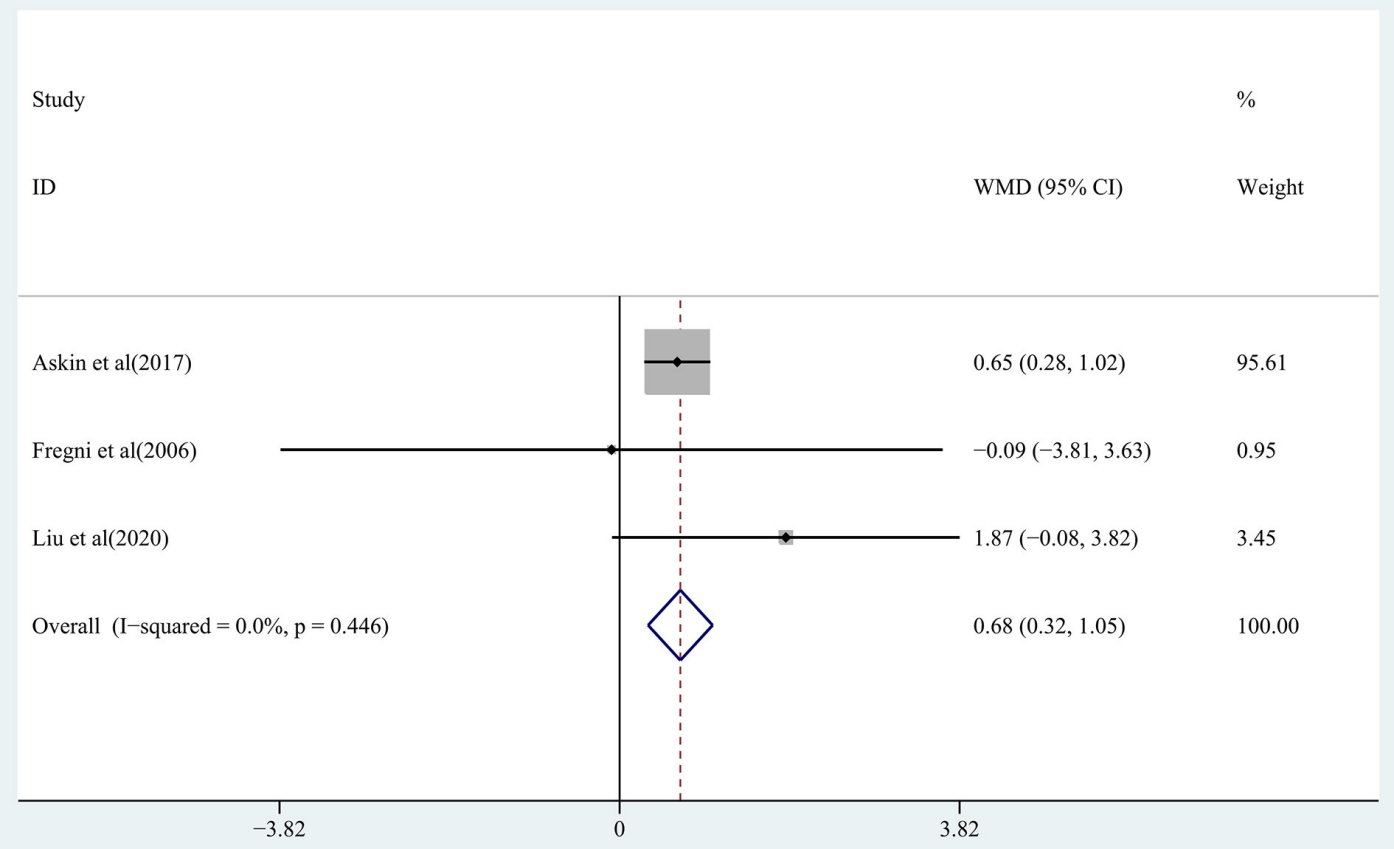
Forest plot of the effect of rTMS treatment on cognitive function recovery in chronic stroke patients.

The effect of RTMS on cognitive function in chronic stroke was assessed by pooling post-intervention data from 3 studies (Fregni et al., 2006; Askin et al., 2017; Liu et al., 2020). Moderate-quality evidence showed a significant improvement in the treatment group (WMD, 0.68; 95% CI: 0.32–1.05; *P* = 0) compared with the control group (Figure 6), with 0% heterogeneity.

### Effect of rTMS on the recovery of psychological disorders in chronic stroke patients

The effect of rTMS on post-stroke depression in patients with chronic-phase stroke was assessed by pooling post-intervention data from four studies (de Oliveira et al., 2014; Gu and Chang, 2017; Sasaki et al., 2017; Hordacre et al., 2021). The pooled data showed no significant reduction in depressive symptoms in the treatment group (SMD: −1.03; 95% CI: −3.06 to 1.00; *P* = 0.32) (Supplementary Figure S3), with high heterogeneity (*I*^2^ = 91.7%, *P* < 0.001).

### Effect of rTMS on the recovery of balance and walking ability in chronic stroke patients

**Figure 7:**
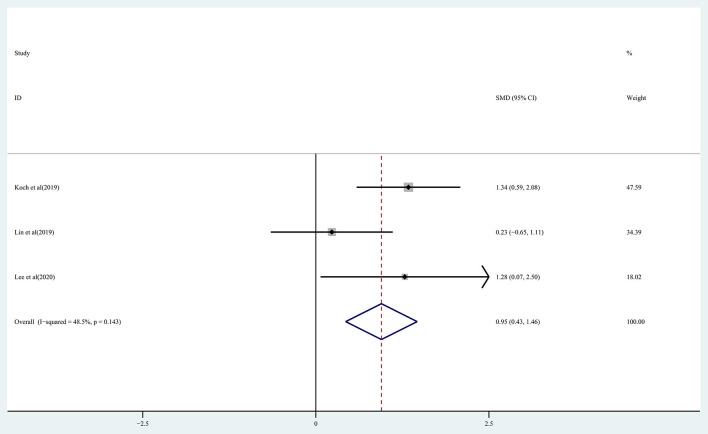
Forest plot of the effect of rTMS treatment on balance function recovery in chronic stroke patients.

The effect of rTMS on balance function in chronic stroke patients was assessed by pooling data from three studies (Koch et al., 2019; Lin et al., 2019; Lee and Cha, 2020). There was moderate quality evidence of a significant improvement in balance in the treatment group (SMD: 0.95; 95% CI: 0.43–1.46; *P* = 0) observed based on the analysis (Figure 7), with heterogeneity (*I*^2^ = 48.5%, *P* = 0.143).

**Figure 8:**
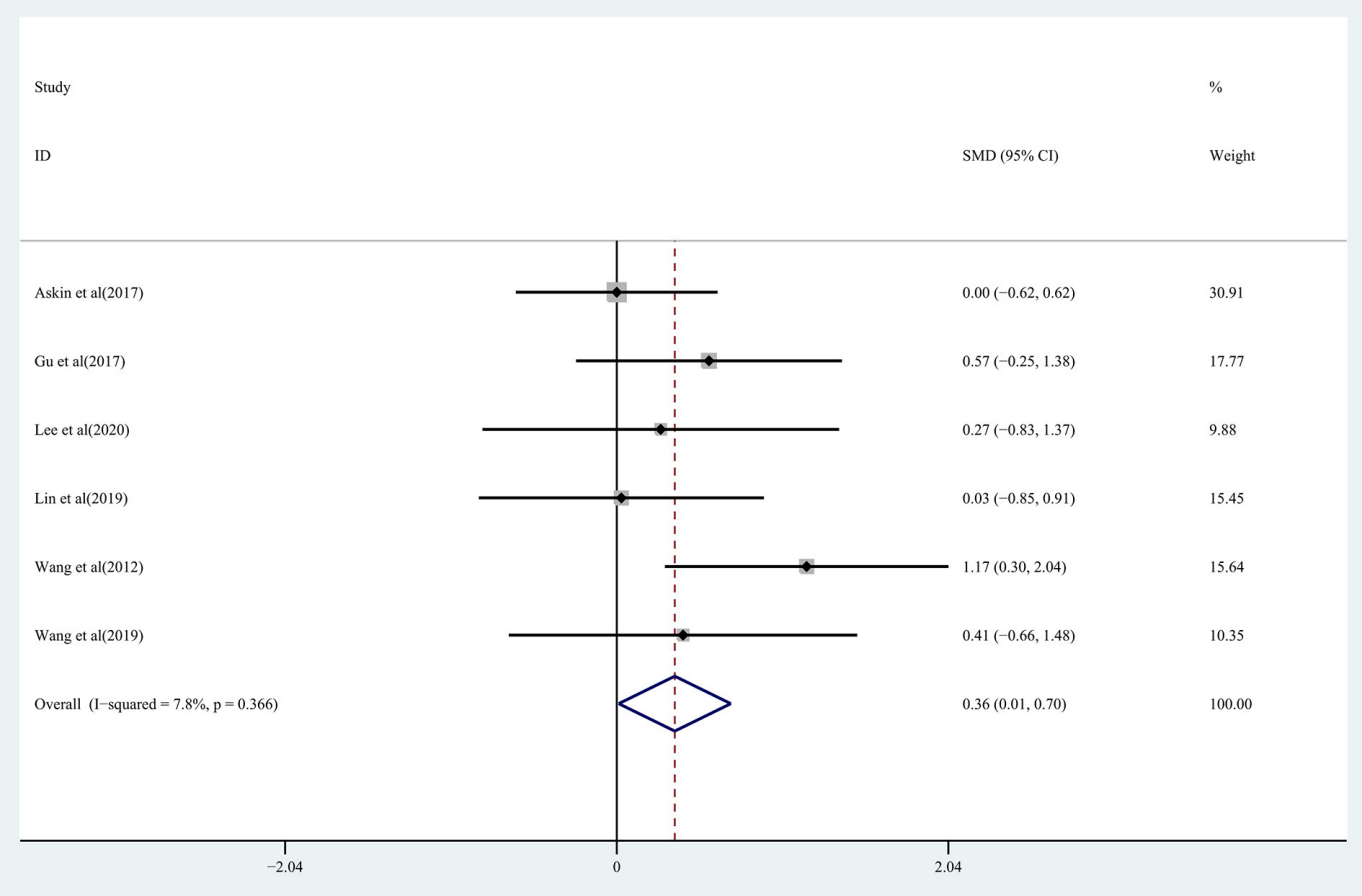
Forest plot of the effect of rTMS treatment on walking function recovery in chronic stroke patients.

Regarding the effect on walking function: the results of the pooled data showed moderate quality evidence of a significant improvement in walking function in the treatment group (SMD: 0.36; 95% CI: 0.01–0.70; *P* = 0.042) (Figure 8), with heterogeneity (*I*^2^ = 7.8%, *P* = 0.366).

### Effect of rTMS on the ability to perform ADLs in chronic stroke patients

**Figure 9:**
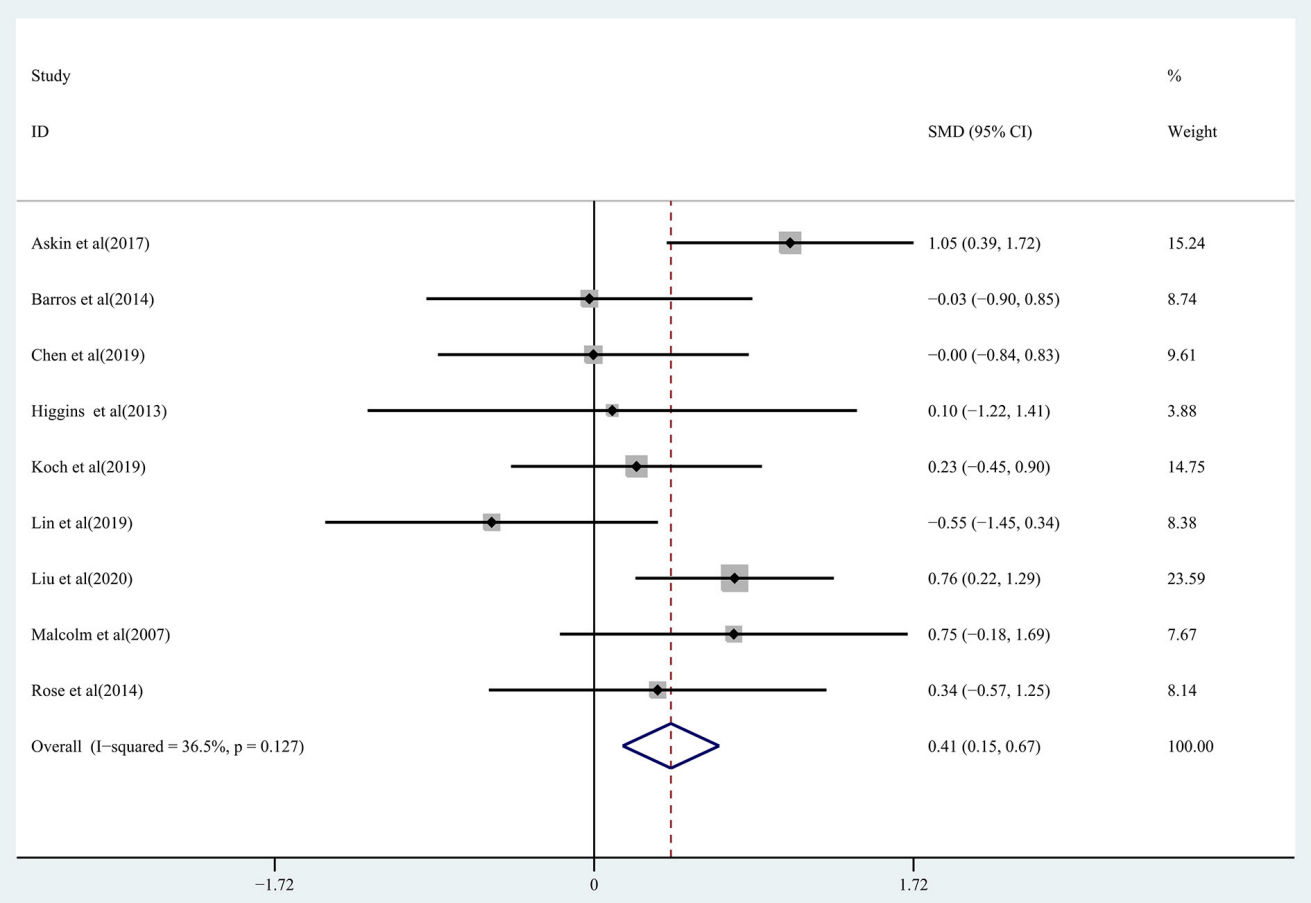
Forest plot of the effect of rTMS treatment on ADL function recovery in chronic stroke patients.

The ability to perform ADLs in chronic stroke patients receiving rTMS treatment was assessed by pooling data from nine studies (Malcolm et al., 2007; Higgins et al., 2013; Barros Galvão et al., 2014; Rose et al., 2014; Askin et al., 2017; Chen et al., 2019; Koch et al., 2019; Lin et al., 2019; Liu et al., 2020). Moderate-quality evidence showed a significant improvement in ADLs in the treatment group (SMD: 0.41; 95% CI: 0.15–0.67; *P* = 0.002) (Figure 9), with heterogeneity (*I*^2^ = 36.5%, *P* = 0.127).

### Sensitivity analysis

Sensitivity analyses were performed on selected studies to identify the potential influence of outliers on the overall results. The results showed no significant influence of any individual study on the results of the meta-analysis (Supplementary Figure S4).

### Adverse event of intervention

Of these studies, 21 monitored for adverse effects and 17 (81%) showed no adverse effects with rTMS. de Oliveira et al. (2014) reported mild headaches observed in 3 patients in the transcranial magnetic stimulation group. Fregni et al. (2006) reported mild headaches in 1 patient and increased anxiety in another patient in the rTMS group. Hordacre et al. (2021) reported two cases of mild neck pain and one case of mild sleep disturbance in the rTMS group. Malcolm et al. (2007) reported no significant adverse effects of rTMS, except for scalp discomfort.

## Discussion

There are currently no meta-analyses regarding rTMS treatment for chronic stroke patients, despite the fact that rTMS and TBS have been utilized to treat post-stroke dysfunction. Thus, the objective of this study was to evaluate the efficacy of rTMS in treating post-stroke pain, balance and walking function, cognitive function, speech and swallowing, motor function, psychological impairment, and ADLs in patients with chronic stroke. The method of assessment was mainly a subjective clinical scale. The results of the analysis revealed that for the motor function in patients with chronic stroke, rTMS significantly improved upper extremity and hand function and reduced muscular tone, but did not significantly improve lower extremities mobility and strength. For other potential sequelae associated with chronic stroke, rTMS significantly improved cognitive function, balance and walking function, and ADLs, but had no significant effect on post-stroke depression. The current study reports the benefits of rTMS on the overall condition of patients with chronic stroke. However, in previous studies (Xu et al., 2021), no significant benefit of rTMS on the modified Ashworth Scale in patients with post-stroke spasticity was observed compared with sham treatment, but the treatment significantly improved stroke patient strength (Zhang et al., 2017b) and upper limb function (Zhang et al., 2017a), which is less consistent with the conclusion derived from our analysis.

In previous studies examining rTMS for upper limb function in patients with chronic stroke (Zhang et al., 2017a), both high-frequency rTMS and low-frequency rTMS were found to significantly improve upper limb function in stroke patients. This contrasts our further subgroup analysis of stimulation frequencies for treating patients with chronic-phase stroke, no significant effect of low-frequency and high-frequency rTMS being found. Subgroup analysis of treatment sites showed that M1 in the affected hemisphere significantly improved upper limb function in patients with chronic-phase stroke compared to M1 in the unaffected hemisphere. McDonnell and Stinear (2017) also found that directly promoting excitability in the affected M1 may be more beneficial than inhibiting excitability in the unaffected M1 to facilitate recovery after stroke. Subgroup analysis of the number of stimulation pulses showed a significantly better effect for 600 pulses than for controls and still no effect when the number of stimulation pulses was >600 pulses. Whereas, the study by Xiang et al. (2019) found no significant difference in recovery of motor function between ≤600 pulses and >600 pulses. The difference in results may be due to the fact that we only included patients with chronic-phase stroke and only had an effect on upper extremity motor function was assessed. We also found that the stimulation pattern for 600 pulses was iTBS or cTBS, whereas the stimulation pattern for >600 pulses was mainly routine repetitive transcranial magnetic stimulation. Therefore, the former stimulation pattern seems to be a more appropriate rTMS treatment strategy for upper limb dysfunction in patients with chronic-phase stroke.

Regarding rTMS for speech and swallowing function in patients with chronic stroke, in a set of studies performed by Barwood et al. (2011a,b), Barwood et al. (2013), 12 patients with chronic non-fluent aphasia post-stroke showed considerable improvements in verbal aspects of speech for naming, describing, and expressing language up to 12 months after 10 interventions of 1 Hz rTMS compared with patients receiving srTMS treatment. Wang et al. (2014) demonstrated significant improvements in conversation, description, and expression of language at 3 months receiving 1 Hz rTMS treatment compared to patients receiving srTMS. In contrast, in a study by Cheng et al. (2017), a 10-day 5 Hz rTMS intervention for dysphagia in chronic stroke patients did not show any significant effect on swallowing function. Transcranial magnetic stimulation may induce cortical plasticity in the human brain networks involved in language function, thus promoting language improvement (Szaflarski et al., 2021). In contrast, for post-stroke dysphagia, early rTMS intervention may better facilitate recovery (Qiao et al., 2022).

For rTMS treatment of cognitive function in chronic stroke patients, a meta-analysis (Hara et al., 2021) published in 2021 found that rTMS significantly improved cognitive function in stroke patients. In the current study, two trials (Fregni et al., 2006; Askin et al., 2017) used 1 Hz rTMS in the unaffected hemisphere primary motor cortex area, and one trial (Liu et al., 2020) used 10 Hz rTMS in the left dorsolateral prefrontal cortex (DLPFC) and reported similar outcomes. However, the evidence regarding rTMS's effect on the cognitive function of chronic stroke patients is still scare and, as a result, more extensive research is required to determine the optimal stimulation parameters.

Post-stroke depression adversely affects the recovery from motor and cognitive deficits following a stroke, and significantly increases the risk of neurovascular events recurring (Das and G. K, 2018), which can be effectively attenuated by rTMS (Shen et al., 2017). In our analysis, four trials employed 10 Hz rTMS, one of which was applied in the dorsal anterior cingulate cortex to the medial prefrontal cortex (Sasaki et al., 2017) and the other three trials were used in the left DLPFC (de Oliveira et al., 2014; Gu and Chang, 2017; Hordacre et al., 2021). The pooled data revealed that rTMS did not significantly improve depressive symptoms in chronic stroke. Given the high heterogeneity of the data (*I*^2^ = 91.7%), we found that three trials had an improving trend for reducing depression, whereas the study conducted by de Oliveira et al. (2014) did not. Further analysis of the available studies revealed that the de Oliveira et al. study used rTMS with a stimulus intensity of 120% RMT, whereas the other three studies used 110% RMT and the difference may be due to the different stimulus intensities. For rTMS intervention in chronic stroke patients with other psychological dysfunctions, a study by Sasaki et al. (2017) found that chronic stroke patients receiving five sessions of high-frequency rTMS from the dorsal anterior cingulate cortex to the medial prefrontal cortex showed considerable improvements in symptoms of apathy. In another study (de Oliveira et al., 2014), a 10-day 10 Hz rTMS intervention in the DLPFC for central post-stroke pain showed no effect on post-stroke anxiety.

Regarding the effects of rTMS treatment on pain in chronic stroke patients, a study by de Oliveira et al. (2014) found no significant improvement in central post-stroke pain with rTMS intervention, whereas a study by Choi and Chang (2018) suggested that 5 Hz rTMS could reduce hemiplegic shoulder pain. In another recent study of 100 patients (Shao, 2021), the authors showed that high-frequency rTMS was clinically effective in improving neck and shoulder pain in hemiplegic patients with chronic stroke.

Our meta-analysis of balance and walking function in chronic stroke patients showed that chronic stroke survivors who received rTMS had considerably better balance and walking ability, which is contrary to a previous meta-analysis (Li et al., 2018) reporting that rTMS significantly improved patients' walking ability, but not their balance function. The conflicting findings may be attributed to the fact that we included other RCTs from recent years and targeted the patient population with a relatively longer disease course (≥6 months).

According to previous studies (Shen et al., 2017), the ADL function of chronic stroke patients can be greatly improved by rTMS. This conclusion was further confirmed by our meta-analysis after the inclusion of more recent RCTs of high methodological quality.

Although most rTMS trials in patients with chronic-phase stroke have demonstrated its effectiveness in promoting functional recovery, the underlying mechanisms by which TMS improves function in stroke patients are not fully understood. As reported by some studies (Yoon et al., 2011; Guo et al., 2017; Luo et al., 2017; Thomson and Sack, 2020), rTMS aids post-stroke recovery by inducing proliferation, migration and neuronal differentiation of neural stem cells around the infarct zone, confers neuroprotection by promoting anti-apoptotic mechanisms in the peri-ischemic region, and reduces post-stroke neuroinflammation by exerting immunomodulatory effects. Improving clinical outcomes through chemotaxis. The other study by Hong et al. (2021) has also shown that high-frequency rTMS intervention in experimental chronic-phase stroke increases the expression of genes related to neurotransmission and neuroplasticity.

Future investigators may consider the importance of conducting large sample, multicenter, high-quality designed clinical trials. More multi-arm randomized controlled trials should be conducted to determine the differences in efficacy of different rTMS approaches in patients with chronic-phase stroke. This will allow for a general consensus to be reached on the application of stimulation parameters (e.g., site of stimulation, mode of stimulation, duration of treatment, dose) for different post-stroke conditions in order to recommend more effective evidence-based interventions. In the future, new technologies such as stem cell therapies and virtual reality technologies that promote neuroregeneration and functional reconstitution, combined with TMS for modulation of brain function, could be used to explore clinically effective multimodal means of promoting neurological rehabilitation in patients with chronic-phase stroke.

Our study has several limitations that must be recognized. Firstly, despite the fact that we conducted a meta-analysis based on various functional aspects in patients with chronic stroke, some functional aspects (such as the recovery of swallowing and speech function after stroke, the improvement of pain after stroke, and aspects of psychological impairment in stroke) only have a small amount of evidence available and therefore cannot be meta-analyzed. Thus, future RCTs are required to provide stronger evidence in these areas. Secondly, due to the small number of studies included in our meta-analysis regarding rTMS treatment for various functional impairments, it was not possible to determine the optimal rTMS stimulation parameters for different functional impairments. Thirdly, more than half of the selected RCTs lacked concealed allocation and blinded therapists, which may lead to subjectivity bias, and the effect of not using concealed allocation may be greater than the effect of valuable interventions. Finally, the protocols included in the studies also revealed notable variances: Due to the wide range of rTMS pulse numbers (from 600 to 3,000), there was a significant heterogeneity in several meta-analyses even though a random-effects model was employed to generalize consistency. Therefore, proper caution should be used when interpreting our findings.

## Conclusion

The current study, which has significance for the rehabilitation of stroke survivors, is the first meta-analysis to date on the efficacy of rTMS on the sequelae of chronic stroke patients. Our results show that rTMS significantly improved upper limb function, hand function, muscle tone, balance and walking function, and ADLs in chronic stroke patients, but not lower limb mobility or strength. Moreover, rTMS also has a considerable positive impact on patients' cognitive performance and apathy, but provides few improvements on post-stroke depression. However, the results of the current study need to be taken carefully due to the small sample size.

## Data availability statement

The original contributions presented in the study are included in the article/Supplementary material, further inquiries can be directed to the corresponding author/s.

## Author contributions

GCh and MW conceived the review and wrote the manuscript. GCh, MW, TL, GCa, and QD researched the literature. YL revised the manuscript for intellectual content. All authors contributed to manuscript revision and read and approved the submitted version.

## Funding

This research was supported by grants 81772438, 81974357, and 82072548 from the National Science Foundation of China and grant 202206010197 from Guangzhou Municipal Science and Technology Program.

## Conflict of interest

The authors declare that the research was conducted in the absence of any commercial or financial relationships that could be construed as a potential conflict of interest.

## Publisher's note

All claims expressed in this article are solely those of the authors and do not necessarily represent those of their affiliated organizations, or those of the publisher, the editors and the reviewers. Any product that may be evaluated in this article, or claim that may be made by its manufacturer, is not guaranteed or endorsed by the publisher.
