# Supplementary material for: Effects of repetitive transcranial magnetic stimulation on sequelae in patients with chronic stroke: A systematic review and meta-analysis of randomized controlled trials
Source: Front Neurosci. 2022 Oct 20;16:998820. doi: 10.3389/fnins.2022.998820 (PMC9630949; doi:10.3389/fnins.2022.998820)
Supplement: Supplementary file 1 [file Data_Sheet_1.docx]

| **Supplementary Table1** Search engine and query | | | | | | | | | | | | |
| --- | --- | --- | --- | --- | --- | --- | --- | --- | --- | --- | --- | --- |
| Search engine | Search query | | | | | | | | | | | Date of search |
| Pubmed (medline) | (((stroke[MeSH Terms]) OR (stroke) OR (cerebrovascular accident)) AND ((transcranial magnetic stimulation[MeSH Terms]) OR (transcranial magnetic stimulation) OR (TMS) OR (repetitive transcranial magnetic stimulation) OR (rTMS) OR (theta burst stimulation) OR (TBS))) AND ((randomized controlled trial[Publication Type]) OR (controlled clinical trial[Publication Type]) OR (randomized[Title/Abstract]) OR (placebo[Title/Abstract]) OR (randomly[Title/Abstract]) OR (trial[Title/Abstract]) OR (groups[Title/Abstract])) | | | | | | | | | | | 11-September-22 |
|  |  |  |  |  |  |  |  |  |  |  |  |  |
|  |  |  |  |  |  |  |  |  |  |  |  |  |
|  |  |  |  |  |  |  |  |  |  |  |  |  |
|  |  | | | | | | | | | | |  |
| Embase | (stroke OR (cerebrovascular AND accident)) AND (tms OR (transcranial AND magnetic AND stimulation) OR (repetitive AND transcranial AND magnetic AND stimulation) OR rtms OR (theta AND burst AND stimulation) OR tbs) AND ('randomized controlled trial':it OR 'controlled clinical trial':it OR randomized:ab,ti OR placebo:ab,ti OR randomly:ab,ti OR trial:ab,ti OR groups:ab,ti) | | | | | | | | | | | 11-September-22 |
|  |  |  |  |  |  |  |  |  |  |  |  |  |
|  |  |  |  |  |  |  |  |  |  |  |  |  |
|  |  |  |  |  |  |  |  |  |  |  |  |  |
|  |  |  |  |  |  |  |  |  |  |  |  |  |
|  |  |  |  |  |  |  |  |  |  |  |  |  |
| Web of science | ((TS=(stroke OR cerebrovascular accident)) AND TS=(transcranial magnetic stimulation OR TMS OR repetitive transcranial magnetic stimulation OR rTMS OR theta burst stimulation OR TBS)) AND TS=(randomized controlled trial OR controlled clinical trial OR randomized OR placebo OR randomly OR trial OR groups) | | | | | | | | | | | 11-September-22 |
|  |  |  |  |  |  |  |  |  |  |  |  |  |
|  |  |  |  |  |  |  |  |  |  |  |  |  |

| **Supplementary Table 2** Characteristics of included studies | | | | | | | | | | | |
| --- | --- | --- | --- | --- | --- | --- | --- | --- | --- | --- | --- |
| **References** | **No. of participants (Exp/Ctr)** | **Mean age (years) (Exp/Ctr)** | **Sex**  **(M/F)** | **Type of Stroke**  **(H/I)** | **Months since**  **Onset**  **(Exp/Ctr)** | **rTMS protocol** | **Sessions** | **Coil***  **/ Location** | **Control**  **condition** | **Outcome measurement** | **Adverse events** |
| Ackerley et al(2016) | 18(9/9) | 61/71 | 12/6 | NA | 20/18 | iTBS,90%AMT,600pulses | 10 | AHM1 | sham coil | ARAT | NO |
| Askin et al (2017) | 40(20/20) | 56.75/58.8 | 29/11 | I:40 | 28.35m/24.35m | 1hz ,90%RMT,1200pulses | 10 | UHM1 | NO | MAS FMA-UE BBT MMSE FIM FAC | NO |
| Barros et al (2014) | 20(10/10) | 57.4/64.6 | 13/7 | 3/17 | 47.8m/58.9m | 1hz ,90%MT,1500pulses | 10 | UHM1 | 2 coils (1  connected and 1  disconnected) | MAS FMA-UE FIM | NO |
| Chen et al (2019) | 22(11/11) | 52.9/52.6 | 14/8 | 17/5 | ≥6m | iTBS,80%AMT,600pulses | 10 | AHM1 | 60%AMT,  coil flipped | MAS FMA-UE BBT MAL | NO |
| de Oliveira et al (2014) | 21(11/10) | 55/57.8 | 10/11 | 4/17 | ≥6m | 10hz,120%RMT,1250pulses | 10 | Left DLPFC | sham coil | HAMD | Mild headache  （Exp:3/Ctr:1） |
| Di Lazzaro et al (2016) | 17(8/9) | 57.88/56.78 | 8/9 | I:17 | 63.25m/61.33m | cTBS.80%AMT,600pulses | 10 | AHM1 | Tilted coil | FMA-UE | NO |
| Di Lazzaro et al (2013) | 12(6/6) | 59.5/57.5 | 7/5 | I:12 | 34.8m/30m | cTBS,80%AMT,600pulses | 10 | AHM1 | sham coil | NHPT ARAT  Grip-Strength | NO |
| Dos Santos et al (2019) | 20(10/10) | 52.4/64.6 | 13/7 | 3/17 | 47.8m/50.1m | 1hz,90%RMT,1500pulses | 10 | UHM1 | sham coil | MAS | NO |
| Fregni et al (2006) | 15(10/5) | 57.70/52.60 | 11/4 | I:15 | 3.52y/3.57y | 1hz,100%MT,1200pulses | 5 | UHM1 | sham coil | MMSE PPT | Mild headache  (Exp:1/Ctr:1)  Anxiety(Exp:1)  Fatigue(Ctr:1) |
| Gu et al (2017) | 24(12/12) | 58.1/58.3 | 11/13 | 7/17 | 10.3m/10.1m | 10hz,110%MT,1000pulses | 10 | Left DLPFC | Tilted coil | FAC BDI HAMD  MI-UE MI-LE | NO |
| Higgins et al (2013) | 9(4/5) | 74/60 | 6/3 | NA | 134m/95m | 1hz,110%MT,1200pulses | 8 | UHM1 | sham coil | BBT Grip-Strength SIS WMFT | NO |
| Hordacre et al (2021) | 11(6/5) | 63.3/61.6 | 9/2 | NA | 5.5Y/3.9Y | 10hz,110%RMT,3000pulses | 10 | Left DLPFC | sham coil | BDI | Neck Pain  (Exp:2/Ctr:2)  Sleep disorders  (Exp:1/Ctr:1) |
| Jeong et al (2020) | 16(8/8) | 54.25/55.23 | 10/6 | 8/8 | 18.45m/19.26m | 10hz,80%RMT,2000pulses | 20 | AHM1 | NA | MFT Grip-Strength | NA |
| Koch et al (2019) | 34(17/17) | 63/65 | 23/11 | I:34 | 14.53m/14.07m | iTBS,80%AMT,1200pulses | 30 | the lateral  cerebellum,contralateral to the affected hemisphere | Tilted coil | BBS BI | NO |
| **Supplementary Table 2** Continued | | | | | | | | | | | |
| **References** | **No. of participants (Exp/Ctr)** | **Mean age (years) (Exp/Ctr)** | **Sex**  **(M/F)** | **Type of Stroke**  **(H/I)** | **Months since**  **Onset**  **(Exp/Ctr)** | **rTMS protocol** | **Sessions** | **Coil***  **/ Location** | **Control**  **condition** | **Outcome measurement** | **Adverse events** |
| Kuzu et al (2021)a | 13(7/6) | 56.3/65 | 6/7 | I:13 | 16.4m/14.5m | 1hz,90%RMT,1200pulses | 10 | UHM1 | sham coil | FMA-UE MAS | NO |
| Kuzu et al (2021)b | 13(7/6) | 61.3/65 | 8/5 | I:13 | 14.5m/14.5m | cTBS,80%AMT,600pulses | 10 | UHM1 | sham coil | FMA-UE MAS | NO |
| Lee et al (2020) | 13(7/6) | 66.85/64.00 | 7/6 | NA | 7.57m/8.16m | 5hz,90%RMT,900pulses | 20 | AHM1 | Tilted coil | 10MWT TUG | NA |
| Lin et al (2019) | 20(10/10) | 60.8/61.1 | 17/3 | 4/16 | 359d/384d | iTBS,100% MT | 10 | Bilateral M1 | sham coil | BBS 10MWT FMA-LE BI | NO |
| Liu et al (2020) | 58(29/29) | 58.55/57.69 | 26/32 | 23/35 | 8.79m/8.62m | 10hz,90%RMT,700pulses | 20 | Left DLPFC | Tilted coil | MMSE FIM | NO |
| Malcolm et al (2007) | 19(9/10) | 68.4/65.7 | 11/8 | 1/18 | 3.9Y/3.8Y | 20hz,90%MT,2000pulses | 10 | AHM1 | sham coil | WMFT MAL BBT | Scalp discomfort |
| Rose et al (2014) | 19(9/10) | 64.7/64.6 | 13/6 | NA | 60.4m/62.8m | 1hz,100%RMT,1200pulses | 16 | UHM1 | sham coil | WMFT FMA-UE GRIP-strength MAS MAL | NO |
| Sasaki et al (2017) | 13(7/6) | 66.1/62.8 | 11/2 | 6/7 | 4.1Y/5.3Y | 10hz,80%RMT,2000pulses | 5 | the region spanning from (dACC) to (mPFC) | sham coil | QIDS | NO |
| Vongvaivanichakul et al (2014) | 14(7/7) | 57.8 | NA | NA | 43m | 1hz,90%RMT,1200pulses | 1 | UHM1 | Tilted coil | WMFT | NA |
| Wang et al (2012) | 24(12/12) | 64.9/62.98 | 15/9 | NA | 1.84Y/2Y | 1hz,90%RMT,600pulses | 10 | UHM1 | Tilted coil | FMA-LE Gait-speed | NO |
| Wang et al (2019) | 14(8/6) | 53.5/54.7 | 11/3 | 8/6 | 31.8m/25.3m | 5hz,90%RMT,900pulses | 9 | AHM1 | Tilted coil | Gait-speed | NO |
| Zhang et al (2022)a | 28(14/14) | 58.21/64.00 | 17/11 | 12/16 | 50.57m/50.86m | cTBS,70%RMT,600pulses +iTBS,70%RMT,600pulses | 10 | Bilateral M1 | 20%RMT | FMA-UE | NA |
| Zhang et al (2022)b | 28(14/14) | 59.50/64.00 | 15/13 | 10/18 | 63.93m/50.86m | iTBS,70%RMT,600pulses | 10 | AHM1 | 20%RMT | FMA-UE | NA |
| Exp, experimental group; Ctr, control group; M, male; F, female; H,Hemorrage; I, Ischemia; RMT, resting motor threshold; AMT, active motor threshold; Hz, hertz; d, days; m, months; y, years; AH, Affected hemisphere; UH, Unaffected hemisphere; M1, primary motor cortex; DLPFC, dorsolateral prefrontal cortex; dACC, dorsal anterior cingulate cortex ; mPFC,medial prefrontal cortex ; ARAT, action research arm test; FMA-UE, Fugl-Meyer Assessment Upper Extremity Scale; WMFT, wolf motor function test; MI-UE, upper extremity Motricity Index; MFT, Manual Function test; FMA-LE, lower extremity Fugl-Meyer Assessment; MI-LE, lower extremity Motricity Index; BBT, Box and Block Test; NHPT, Nine Hole Peg Test; PPT, Purdue Pegboard Test; MAS, modified Ashworth Scale; TUG, time up and go test; BBS, berg balance scale; FAC, functional ambulation category; 10MWT, 10-meter Walk Test; MMSE, mini mental status examination; BDI, beck depression inventory; HAMD, Hamilton Rating Scale for Depression; QIDS, quick inventory of depressive symptomatology; FIM, Functional Independence Measure; BI, Barthel Index; MAL, Motor Activity Log; SIS, stroke impact scale; NA, not available. | | | | | | | | | | | |

| **Supplementary Table 3**  Grade (summary of findings) | | | | | | |
| --- | --- | --- | --- | --- | --- | --- |
| **rTMS group compared to control group for chronic stroke patients**  **Patient or population:** chronic stroke patients **Settings:**  **Intervention:** rTMS group **Comparison:** control group | | | | | | |
| **Outcomes** | **Illustrative comparative risks* (95% CI)** | | **Relative effect (95% CI)** | **No of Participants (studies)** | **Quality of the evidence (GRADE)** | **Comments** |
|  | Assumed risk | Corresponding risk |  |  |  |  |
|  | **Control group** | **rTMS group** |  |  |  |  |
| **UE function** |  | The mean function in the intervention groups was **0.49 standard deviations higher** (0.08 to 0.89) |  | 280 (15 studies) | ⊕⊕⊝⊝ **low**^1,2^ | SMD 0.49 (0.08 to 0.89) |
| **Hand function** |  | The mean function in the intervention groups was **0.45 standard deviations higher** (0.08 to 0.83) |  | 117 (6 studies) | ⊕⊕⊕⊝ **Moderate** ^2^ | SMD 0.45 (0.08 to 0.83) |
| **Muscle tone** |  | The mean muscle tone in the intervention groups was **0.37 standard deviations lower** (0.51 to 0.24 lower) |  | 139 (7 studies) | ⊕⊕⊕⊝ **Moderate** ^2^ | WMD -0.37 (-0.51 to -0.24) |
| **Cognetic function** |  | The mean function in the intervention groups was **0.68 standard deviations higher** (0.32 to 1.05) |  | 113 (3 studies) | ⊕⊕⊕⊝ **Moderate** ^2^ | WMD 0.68 (0.32 to 1.05) |
| **Balance function** |  | The mean function in the intervention groups was **0.95 standard deviations higher** (0.43 to 1.46) |  | 67 (3 studies) | ⊕⊕⊕⊝ **Moderate** ^2^ | SMD 0.95 (0.43 to 1.46) |
| **Walking function** |  | The mean function in the intervention groups was **0.36 standard deviations higher** (0.01 to 0.70) |  | 135 (6 studies) | ⊕⊕⊕⊝ **Moderate** ^2^ | SMD 0.36 (0.01 to 0.70) |
| **ADLs** |  | The mean ADL in the intervention groups was **0.41 standard deviations higher** (0.15 to 0.67) |  | 241 (9 studies) | ⊕⊕⊕⊝ **Moderate** ^2^ | SMD 0.41 (0.15 to 0.67) |
| *The basis for the **assumed risk** (e.g., the median control group risk across studies) is provided in footnotes. The **corresponding risk** (and its 95% confidence interval) is based on the assumed risk in the comparison group and the **relative effect** of the intervention (and its 95% CI). **CI:** Confidence interval; **UE:** Upper extremity | | | | | | |
| GRADE Working Group grades of evidence **High quality:** Further research is very unlikely to change our confidence in the estimate of effect.  **Moderate quality:** Further research is likely to have an important impact on our confidence in the estimate of effect and may change the estimate. **Low quality:** Further research is very likely to have an important impact on our confidence in the estimate of effect and is likely to change the estimate. **Very low quality:** We are very uncertain about the estimate. | | | | | | |
| ^1^ Serious inconsistency due to moderate heterogeneity with 50% < I ^2^ < 75% and P value (chi-square test) < 0.05. ^2^ Very serious imprecision due to the small sample size (< 400 individuals) and wide confidence interval | | | | | | |


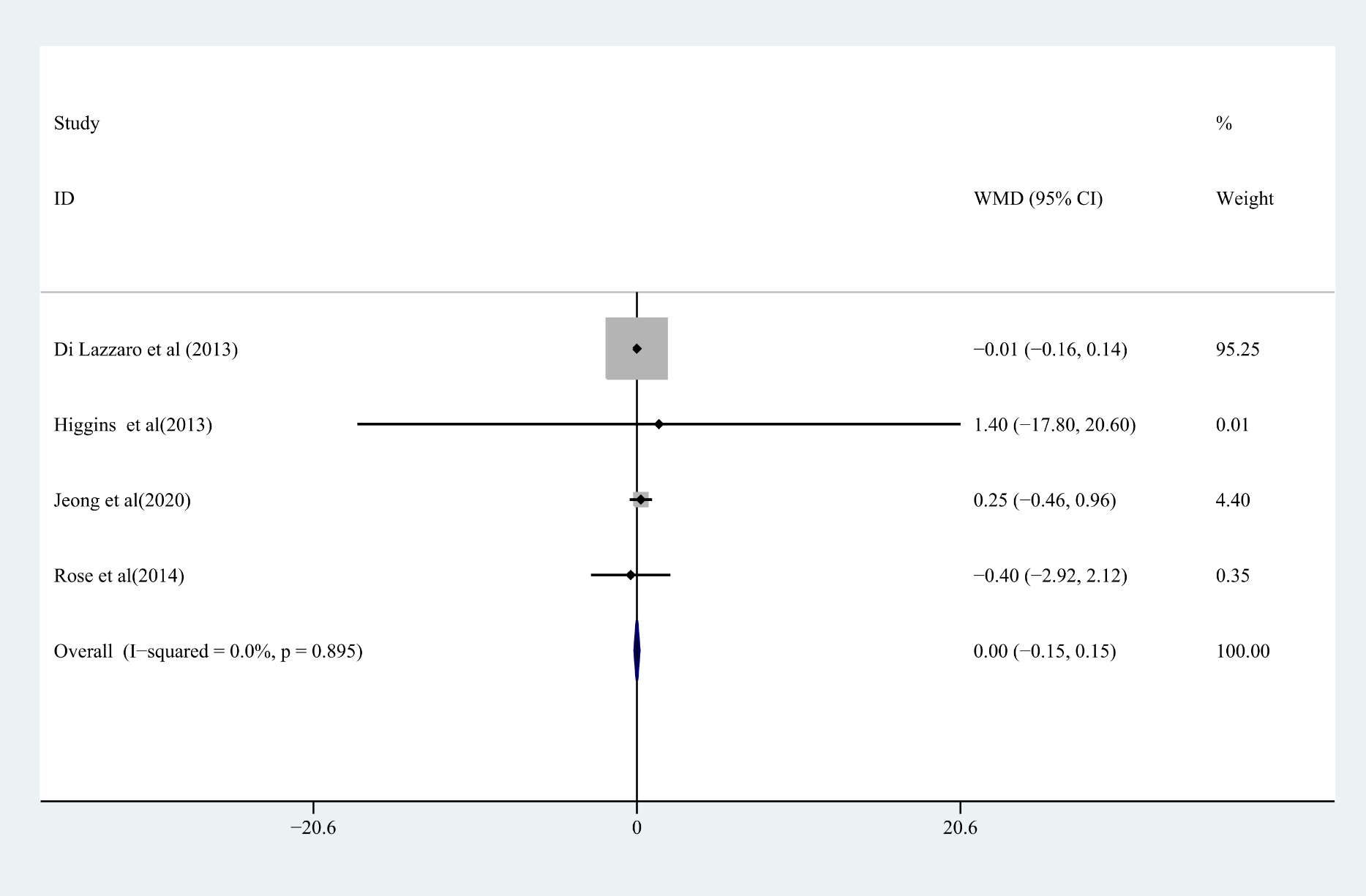


**Supplementary Figure 1** Forest plot of the effect of rTMS treatment on strength recovery in chronic stroke patients.

**Supplementary Figure 2** a. Forest plot of upper extremity function recovery in chronic stroke patients disaggregated by stimulation frequency compared with controls; b. Forest plot of upper extremity function recovery in chronic stroke patients disaggregated by treatment site compared with controls; c. Forest plot of upper extremity function recovery in chronic stroke patients disaggregated by number of stimulation pulses compared with controls.


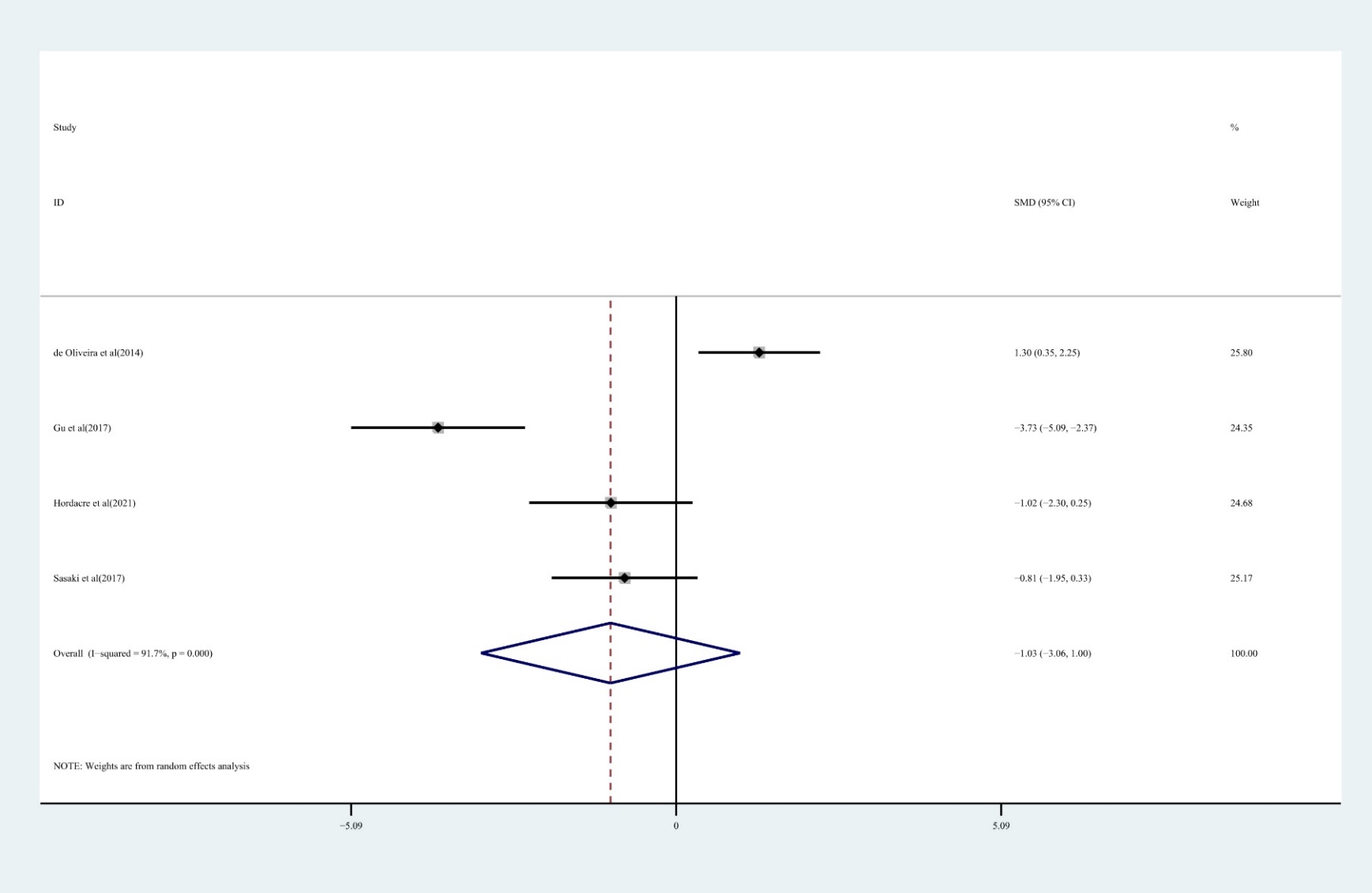


**Supplementary Figure 3** Forest plot of the effect of rTMS treatment on depression reduction in chronic stroke patients.


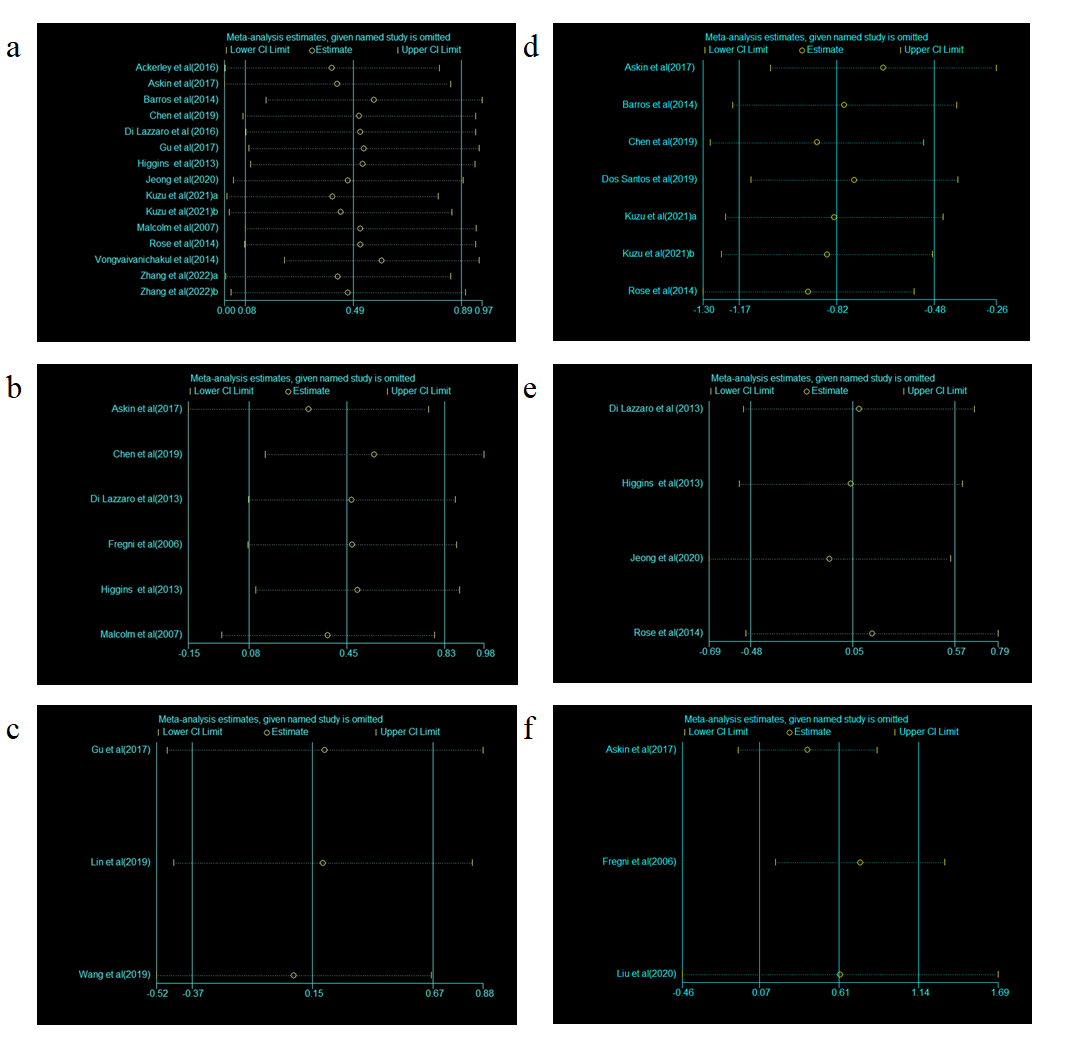


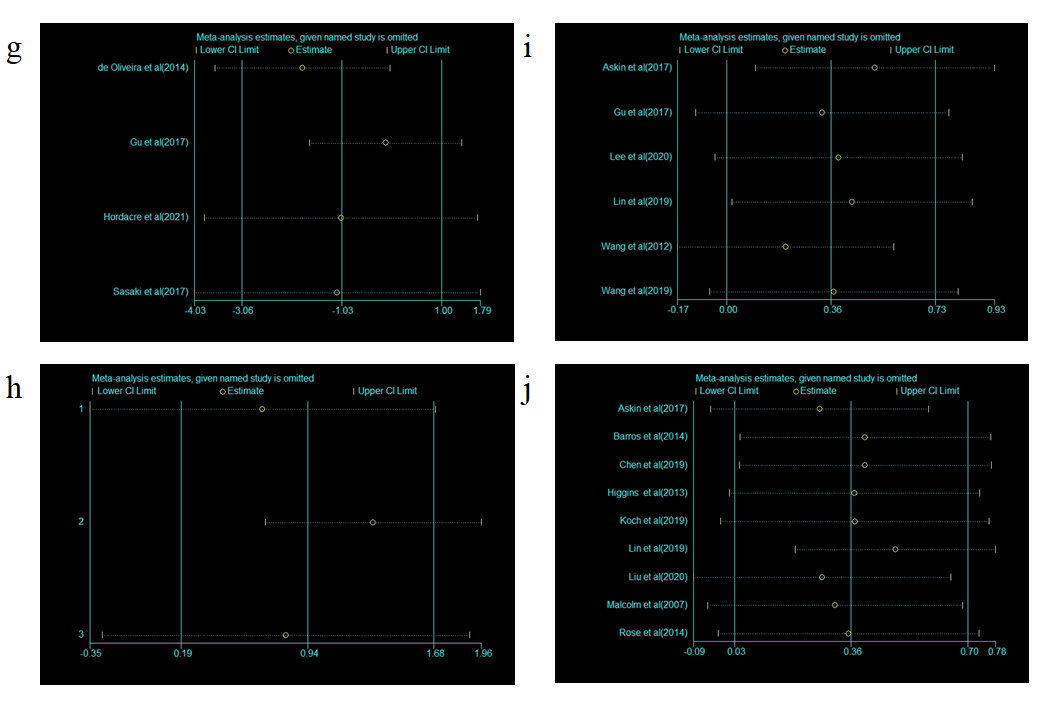


**Supplementary Figure 4** Results of sensitivity analysis showing the stability of the results in the included studies: a. upper extremity motor function; b. hand function; c. lower extremity motor function; d. muscle tone; e. strength; f. cognitive function; g. depressive symptoms; h. balance function; I. walking function; j. ADL function.
